# Early-Onset Dialysis Disequilibrium Syndrome Presenting With Hypoxia and Subtle Neurological Signs During Initial Hemodialysis

**DOI:** 10.7759/cureus.108587

**Published:** 2026-05-10

**Authors:** Muhammad Hasnain Tahir, Fatima Tahir, Samina Asghar

**Affiliations:** 1 Cardiovascular Medicine, Stanford Health Care, San Francisco, USA; 2 Cardiology, Stanford University Medical Center, Stanford, USA; 3 Obstetrics and Gynaecology, Gujranwala Medical College, Gujranwala, PAK

**Keywords:** case report, cerebral edema, dialysis disequilibrium syndrome, hemodialysis, hemodialysis complications, hypoxia, severe uremia

## Abstract

Dialysis disequilibrium syndrome (DDS) is a rare but serious neurological complication seen in patients with severe uremia during initiation of hemodialysis. Early recognition is essential to prevent morbidity.

We report a 35-year-old male with obstructive uropathy presenting with severe azotemia (urea = 316 mg/dL, creatinine = 24 mg/dL). He underwent his first hemodialysis session, which was conducted over 2.5 hours using a low-efficiency approach. Within 15-30 minutes of initiation, he developed vomiting, decreased responsiveness, and staring spells without overt seizures. Oxygen saturation dropped to 80%, requiring supplemental oxygen.

Due to an altered mental status, subjective symptoms could not be assessed at presentation. A clinical diagnosis of DDS was strongly considered based on temporal association and risk profile. The patient improved within 12 hours with supportive care. Subsequent dialysis sessions were modified to a low-efficiency strategy and were well tolerated.

This case highlights the importance of recognizing atypical neurological presentations of DDS, particularly in patients with impaired communication, and emphasizes preventive dialysis strategies.

## Introduction

Dialysis disequilibrium syndrome (DDS) is a neurological complication associated with hemodialysis, most commonly occurring during initial treatment in patients with severe uremia. Although less common with modern dialysis practices, it remains an important clinical consideration in high-risk individuals [1].

DDS is believed to result from rapid osmotic shifts between plasma and brain tissue during dialysis, leading to cerebral edema. Clinical manifestations range from mild symptoms such as nausea and headache to severe neurological impairment, including seizures and coma.

Recognition of DDS may be challenging in patients who cannot communicate symptoms effectively or present with an altered mental status. This case illustrates an atypical presentation of suspected DDS with subtle neurological findings and hypoxia during initial hemodialysis.

## Case presentation

A 35-year-old male with a history of obstructive uropathy presented with severe renal dysfunction, including serum urea of 316 mg/dL (reference range = 30-45 mg/dL) and serum creatinine of 24 mg/dL (reference range = 0.6-1.2 mg/dL). On admission, his blood pressure was 200/100 mmHg, heart rate was 120 beats per minute, respiratory rate was 14 breaths per minute, and temperature was within normal limits. The patient was unable to provide a reliable history due to an altered mental status at presentation.

Given severe uremia, he was initiated on his first hemodialysis session, conducted for approximately 2.5 hours using a low-efficiency protocol. Within 15-30 minutes of initiation, he developed repeated vomiting, decreased responsiveness, and staring spells. No generalized tonic-clonic seizures were observed.

During this episode, oxygen saturation dropped to 80%, requiring supplemental oxygen at 4 liters per minute. The hypoxia was transient and likely related to hypoventilation and possible aspiration risk in the setting of altered mental status.

Based on the temporal relationship between dialysis initiation and symptom onset in the setting of severe uremia, DDS was strongly suspected. However, alternative diagnoses, including uremic encephalopathy and delirium, were also considered. Neuroimaging was not performed.

The patient was managed conservatively with supportive care, including close monitoring and appropriate fluid and hemodynamic management. His symptoms gradually improved, and he returned to baseline within approximately 12 hours.

The patient also had severe anemia, with a hemoglobin level of 3.9 g/dL (reference range = 13.5-17.5 g/dL), attributed to chronic kidney disease secondary to obstructive uropathy, and was managed with transfusion support.

For subsequent sessions, dialysis was modified to a low-efficiency approach, with a duration of 2.5 hours and reduced blood flow rates to allow gradual solute removal. These sessions were well tolerated without recurrence of symptoms.

## Discussion

DDS is an uncommon but clinically significant complication of hemodialysis, typically occurring during initial sessions in patients with severe uremia [1]. It represents a spectrum of neurological dysfunction ranging from mild symptoms to severe manifestations such as seizures and coma.

The pathophysiology is primarily related to rapid osmotic shifts between plasma and the central nervous system. Rapid removal of urea from plasma during hemodialysis creates an osmotic gradient, leading to water movement into brain cells and resulting in cerebral edema [2,3]. Additional mechanisms, such as intracellular acidosis and blood-brain barrier alterations, have also been proposed.

A key feature of this case is its atypical clinical presentation in the setting of significant diagnostic limitations due to impaired communication at baseline. Because the patient was unable to provide a reliable history, subjective symptoms such as headache, dizziness, or confusion could not be elicited, and clinical assessment relied entirely on objective findings. These included repeated vomiting, reduced responsiveness, and staring spells without overt convulsive activity, which are suggestive of early neurological involvement during the dialysis session.

The presence of transient hypoxia further added complexity to the clinical picture. Although hypoxia is not a classical feature of DDS, it may be explained in this context by hypoventilation, transient respiratory compromise, or possible aspiration risk in the setting of acute neurological deterioration. This finding highlights the importance of close cardiorespiratory monitoring during initial hemodialysis sessions in high-risk patients [4].

Importantly, a broad differential diagnosis was considered, including uremic encephalopathy, acute electrolyte disturbances, and delirium related to severe systemic illness. However, the close temporal relationship between initiation of hemodialysis and onset of symptoms, combined with the patient’s significant risk factors, including severe uremia and first exposure to dialysis, and the subsequent rapid clinical recovery following supportive management, made DDS the most likely clinical diagnosis. Neuroimaging was not performed in this case; therefore, radiological confirmation of cerebral edema could not be obtained, and the diagnosis remained clinical in nature.

Additionally, severe anemia may have further reduced physiological reserve and potentially increased susceptibility to neurological instability during rapid osmotic shifts associated with initial dialysis. In response to this event, subsequent hemodialysis sessions were modified to a low-efficiency strategy with gradual solute removal, which is a well-established preventive approach to reduce the risk of recurrence of DDS in high-risk patients.

Overall, this case underscores the importance of early recognition of subtle neurological and systemic changes during initial hemodialysis, particularly in patients with communication barriers, and reinforces the need for individualized, low-efficiency dialysis strategies to improve safety and prevent neurological complications.

## Conclusions

DDS should be considered in patients with severe uremia who develop acute neurological symptoms shortly after initiation of hemodialysis, even when classical features are absent or subtle. This case emphasizes that diagnosis can be particularly challenging in patients with impaired communication, where reliance on objective clinical findings and temporal association becomes essential. The overlap of clinical features with other conditions, such as uremic encephalopathy, delirium, and metabolic disturbances, highlights the importance of maintaining a broad differential diagnosis in similar clinical scenarios. Although neuroimaging was not performed, the clinical course and rapid recovery following supportive management were most consistent with DDS.

This case further reinforces the importance of preventive strategies, particularly the use of low-efficiency, carefully titrated initial hemodialysis sessions in high-risk patients with severe azotemia. Early recognition and appropriate modification of dialysis parameters remain critical to preventing potentially serious but reversible neurological complications.

## References

[1] Kennedy AC, Linton AL, Eaton JC (1962). Urea levels in cerebrospinal fluid after haemodialysis. Lancet.

[2] Arieff AI (1994). Dialysis disequilibrium syndrome: current concepts on pathogenesis and prevention. Kidney Int.

[3] Silver SM (1995). Cerebral edema after rapid dialysis is not caused by an increase in brain organic osmolytes. J Am Soc Nephrol.

[4] Mistry K (2019). Dialysis disequilibrium syndrome prevention and management. Int J Nephrol Renovasc Dis.

